# Temporal evolution of sulfadoxine-pyrimethamine resistance genotypes and genetic diversity in response to a decade of increased interventions against *Plasmodium falciparum* in northern Ghana

**DOI:** 10.1186/s12936-021-03693-3

**Published:** 2021-03-17

**Authors:** Lucas N. Amenga-Etego, Victor Asoala, Godfred Agongo, Christopher Jacob, Sonia Goncalves, Gordon A. Awandare, Kirk A. Rockett, Dominic Kwiatkowski

**Affiliations:** 1 West African Centre for Cell Biology of Infectious Pathogens, Department of Biochemistry, Cell and Molecular Biology, University of Ghana, Legon, Accra, Ghana; 2 Navrongo Health Research Centre, Ghana Health Service, P. O. Box 114, Navrongo, Ghana; 3 Wellcome Centre for Human Genetics, University of Oxford, Headington, Oxford, OX3 7BN UK; 4 Wellcome Sanger Institute, Wellcome Genome Campus, Hinxton, Cambridgeshire UK; 5 Big Data Institute, University of Oxford, Oxford, UK

**Keywords:** Malaria, SP resistance, Genotypes, Haplotype, *Pfdhfr*, *Pfdhps*, Temporal

## Abstract

**Background:**

Anti-malarial drug resistance remains a key concern for the global fight against malaria. In Ghana sulfadoxine-pyrimethamine (SP) is used for intermittent preventive treatment of malaria in pregnancy and combined with amodiaquine for Seasonal Malaria Chemoprevention (SMC) during the high malaria season. Thus, surveillance of molecular markers of SP resistance is important to guide decision-making for these interventions in Ghana.

**Methods:**

A total of 4469 samples from uncomplicated malaria patients collected from 2009 to 2018 was submitted to the Wellcome Trust Sanger Institute, UK for DNA sequencing using MiSeq. Genotypes were successfully translated into haplotypes in 2694 and 846 mono infections respectively for *pfdhfr* and *pfdhps genes* and the combined *pfhdfr/pfdhps* genes across all years.

**Results:**

At the *pfdhfr* locus, a consistently high (> 60%) prevalence of parasites carrying triple mutants (**IRN**I) were detected from 2009 to 2018. Two double mutant haplotypes (N**RN**I and **I**C**N**I) were found, with haplotype N**RN**I having a much higher prevalence (average 13.8%) than **I**C**N**I (average 3.2%) across all years. Six *pfdhps* haplotypes were detected. Of these, prevalence of five fluctuated in a downward trend over time from 2009 to 2018, except a *pfdhps* double mutant (**AG**KAA), which increased consistently from 2.5% in 2009 to 78.2% in 2018. Across both genes, *pfdhfr/pfdhps* combined triple (N**RN**I + **A**AKAA) mutants were only detected in 2009, 2014, 2015 and 2018, prevalence of which fluctuated between 3.5 and 5.5%. The combined quadruple (**IRN**I + **A**AKAA) genotype increased in prevalence from 19.3% in 2009 to 87.5% in 2011 before fluctuating downwards to 19.6% in 2018 with an average prevalence of 37.4% within the nine years. Prevalence of parasites carrying the quintuple (**IRN**I + **AG**KAA or S**GE**AA) mutant haplotypes, which are highly refractory to SP increased over time from 14.0% in 2009 to 89.0% in 2016 before decreasing to 78.9 and 76.6% in 2017 and 2018 respectively. Though quintuple mutants are rising in prevalence in both malaria seasons, together these combined genotypes vary significantly within season but not between seasons.

**Conclusions:**

Despite high prevalence of *pfdhfr* triple mutants and combined *pfdhfr/pfdhps* quadruple and quintuple mutants in this setting SP may still be efficacious. These findings are significant as they highlight the need to continuously monitor SP resistance, particularly using deep targeted sequencing to ascertain changing resistance patterns.

**Supplementary Information:**

The online version contains supplementary material available at 10.1186/s12936-021-03693-3.

## Background

Malaria disproportionately leads to childhood morbidity and mortality in sub-Saharan Africa (sSA). An estimated 24 million children were infected with *Plasmodium falciparum* in 2018 in sSA with Ghana being one of top ten countries in Africa with the highest absolute increases in cases of malaria in 2018 compared to the previous year [1]. Due to persistent high malaria transmission, the Ghana National Malaria Control Programme (NMCP) prioritized the northern regions for high-impact interventions, such as indoor residual spraying (IRS), seasonal malaria chemoprevention (SMC) among children under 5 years old [2], increased coverage of long-lasting insecticidal bed nets (LLINs) and intermittent preventive treatment in pregnancy (IPTp) [3, 4]. The World Health Organization (WHO) recommends use of SP plus amodiaquine (SP-AQ) for SMC in areas of high seasonal malaria transmission in the Sahel sub-region of Africa [5]. Therefore, despite the withdrawal of SP in 2005 as a first-line anti-malarial in Ghana [6], it remains a key component in interventions targeting vulnerable groups such as pregnant women and young children. However, the success of targeted interventions such as, IPTp and SMC, is largely dependent on the population prevalence of bifunctional dihydrofolate reductase-thymidylate synthase (*dhfr)* and dihydropteroate synthetase (*dhps)* SP resistance mutations [7–9].

Resistance of *P. falciparum* to SP is caused by well documented single nucleotide polymorphisms (SNPs) in the *pfdhfr* and *pfdhps* genes that encode enzymes in the folate metabolism pathway and are targeted by pyrimethamine and sulfadoxine, respectively [10, 11]. A change from wild-type Ser108 to Asn108 (S108N) in *pfdhfr* is associated with low level pyrimethamine tolerance both in vitro and in vivo [10–12]*.* This basal S108N amino acid substitution is characterized to have a tenfold increased risk of SP therapeutic failure [13]. The progressive accumulation of other mutations that result in altered amino acid substitutions including *pfdhfr*-C50R, *pfdhfr*-N51I, *pfdhfr*-C59R, and *pfdhfr*-I164L that can result in higher levels of resistance to pyrimethamine and diminish the efficacy of SP in vivo [14]. Parasites carrying multiple mutations in the *pfdhfr* gene have evolved independently in different populations throughout the malaria endemic world [15], and these haplotypes are often associated with higher levels of resistance as compared to single mutant genotypes. Typically, the most prevalent multi-locus genotype found in areas of high SP resistance in sSA is the triple *pfdhfr* mutant genotype (*pfdhfr*-51I/59R/108N/I164) [16, 17]. A quadruple *pfdhfr* mutant first detected in southeast Asia in the late 1980s, includes an additional I164L substitution [18]. Parasites carrying the quadruple haplotype are associated with high SP treatment failure [19]. Thus far, parasites carrying this allele have been only detected in parts of East Africa [16, 20], but not West Africa [11, 12].

Parasites carrying the triple *pfdhr* mutant (108N/51I/59R/I164) alleles and additional *pfdhps* double mutant (G437/540E) alleles have been associated with strong resistance to SP [21]. A further mutation at codon 581 of *pfdhps* results in a triple mutant allele (A437G/K540E/A581G), which in combination the *pfdhfr* triple mutant confers a high rate of SP treatment failure [22, 23]. The occurrence of the K540E mutation is a proxy marker for a variant quintuple mutant genotype containing both the *pfdhps* double mutant (A437G/K540E) and the *pfdhfr* triple mutant genotypes, which is also highly correlated with SP treatment failure in children [9, 21, 22].

The objectives of the present study were to determine the temporal trends in prevalence of multi-locus SP resistance markers in *P. falciparum* isolates collected from clinical sources between 2009 and 2018, a period spanning major interventions for reducing the burden of malaria and disrupting transmission including IRS, SMC and IPTp. This study also explores the seasonal risk of carrying SP resistant genotypes in northern Ghana.

## Methods

### Study site

**Fig. 1:**
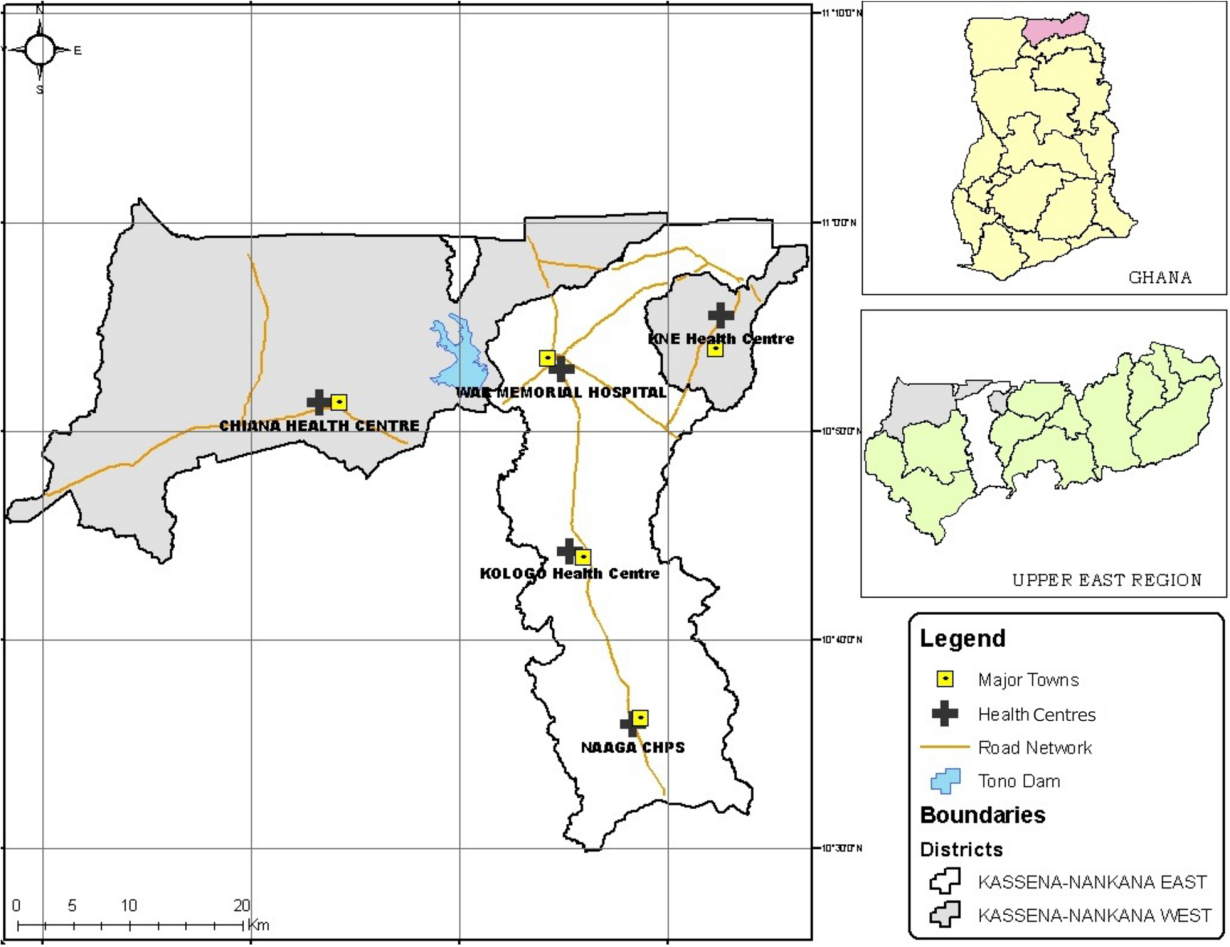
Location of the Upper East Region in Ghana, The Kassena-Nankana Districts in the Upper East Region and Kassena-Nankana East and West Districts showing location of Health facilities from which samples were collected. CHPS; Community Health and Planning Services

The study was conducted in the Kassena-Nankana East Municipality and Kassena-Nankana West District, two adjoining administrative areas in the Upper East Region of northern Ghana, here referred to as KNDs. The KNDs cover a total area of about 1675km^2^ and lie between latitude 10.30′ and 11.10′ North and longitude 1.1′ West close to the Ghana-Burkina Faso border (Fig. 1). Average rainfall is estimated at about 1300 mm, but this has been consistently dwindling in recent years [24]. The KNDs population is estimated at 160,000 inhabitants with a population density of 91.5 per square kilometre [25]. The proportion of children under 5 years has been estimated at about 11.0% and there are approximately 4000 births per year. The KNDs population is under continuous demographic surveillance by the Navrongo Health and Socio-demographic Surveillance System. There is one main hospital, the Navrongo War Memorial Hospital (NWMH) that serves as a referral unit for the population, nine satellite clinics and several additional community-based health planning and services compounds strategically located to increase access to basic primary health care [26]. Malaria transmission is intense with seasonal fluctuations largely dependent on the rainfall pattern [24, 25]. The high transmission season coincides with the short rainy season from July to November, while the low season occurs during the dry months of December to May. The main malaria vector is *Anopheles gambiae* and the annual entomological inoculation rate (EIR) was estimated to vary between 1132 and 157 infective bites/person/year in the KNDs [27].

### Ethics statement

Scientific and ethical clearance was obtained from the Navrongo Health Research Centre Institutional Review Board (# NHRCIRB203). Informed consent was obtained from all individuals or their parents/guardians prior to enrollment into each study.

### Study procedure

All-age patients with fever (or history of fever within the past 24hrs) reporting to the NWMH, and four clinics in the KNDs were screened for malaria from January to December of each year from 2009 to 2018, except 2012 when no survey was conducted. *Plasmodium falciparum* Histidine Rich Protein 2-based rapid diagnostic test (RDT)[CareStart™ malaria Pf (HRP2), Access Bio, NJ, USA] was used. Informed consent was documented for RDT positive patients and those willing to participate were enrolled. Four 50 µL dried blood spots (DBS) and malaria smears were prepared from a finger prick. *Plasmodium falciparum* density was determined by microscopy and asexual parasites scored against 200 white blood cells (WBCs) and smears were considered negative after examining 100 fields without detecting asexual or sexual parasites. Parasite density per 200 WBCs was converted to density per microlitre assuming 8000 WBCs.

Parasite DNA was extracted from DBS samples using the QIAamp DNA Midi kit (Qiagen, UK) as per the manufacturer's protocol.

### Genotyping of Drug resistance markers

Samples between the 2009 and 2013 studies were submitted to the Malaria Genomic Epidemiology Network (MalariaGEN; http://www.malariagen.net/) at the Wellcome Sanger Institute, UK for whole genome sequencing as previously described [28]. Genotypes were assigned for variants with a read depth ≥ 5. Genotypes for the selected resistance genes were then extracted, translated and annotated into known SP resistance markers. For samples collected between 2014 and 2018, amplicon sequencing of SP resistance markers was performed by MalariaGEN using an amplicon-sequencing protocol (https://www.medrxiv.org/content/10.1101/2020.07.23.20159624v1 and https://www.malariagen.net/resource/29). In brief, locus-specific multiplexed primers were designed using MPprimer software [29] with some modifications. Primers were designed to generate amplicons each of between 190 and 250 bp and were assigned to one of three pools. A two-step PCR protocol was used for each pool to first amplify the regions of interest in the parasite genome, followed by a second PCR to incorporate sequencing and unique sample-level and primer-pool multiplexing adapters. Multiple samples were sequenced on a single MiSeq lane combining all 3 pools of the PCR amplicons. Sequenced samples were de-plexed using the unique multiplexing adapter IDs and the individual sample-level CRAM files were aligned to a modified amplicon *P. falciparum* reference genome. Genotyping of bi-allelic SNPs was undertaken using bcftools as well as custom scripts to determine genotypes which were translated and annotated into known drug resistance haplotypes (https://www.medrxiv.org/content/10.1101/2020.07.23.20159624v1 and https://www.malariagen.net/resource/29).

### Within-host diversity estimation

Within-host diversity of (complexity) infections was determined using either the genome-wide *Fws metric* in the 2009 to 2013 sequence data or *THE REAL McCOIL* to estimate infection complexity in the 2014 to 2018 amplicon data. The McCOIL’s categorical method, which uses likelihood estimation to determine the number of distinct parasite genome-wide haplotypes (strains) within each sample was implemented [30].

### Haplotype reconstruction

Owing to the high within host diversity, in the 2009 to 2013 collections, genotypes were translated into haplotypes for each isolate based on the major allele for *pfdhfr* and *pfdhps* loci. The amplicon data from 2014 to 2018 however, enabled the genotyping of pure or mixed (heterozygous) samples and the reconstruction of haplotypes for both pure and heterozygous samples, accounting for both major and minor clones in mixed infections. Only samples with Fws > 0.95 and MOI = 1 were used for construction of haplotypes across both genes to obtain *pfdhfr/pfdhps* combined genotypes.

### Data and statistical analysis

To determine the temporal trends of multi-locus SP resistance markers, a year-on-year fluctuation in SP resistance markers was examined. To further explore the effect of malaria interventions, the data was grouped into three windows to reflect periods of major interventions-2009–2011(pre-IRS and SMC), 2013–2015 (IRS) and 2016–2018 (SMC). However, it is worth noting that IPTp and bednets were implemented across all study periods. The following analysis approaches were explored: a descriptive analysis of temporal trends in proportion of isolates with SP resistant marker haplotypes, mixed species infections and within-host diversity of infections. The χ^2^ test was used to compare categorical variables among groups. The non-parametric Kruskal–Wallis test was used for group comparisons as appropriate for the distribution. All statistical tests were two-tailed and statistical significance set at p < 0.05. Data were analysed using the open source statistical software R version 3.4.1.

## Results

A total 4469 patients with uncomplicated malaria were enrolled into this study in the years 2009–2018. Of these patients, 2694 [(2009; n = 120); (2010; n = 184); (2011; n = 55); (2013; n = 33); (2014; n = 323); (2015; n = 178); (2016; n = 920); (2017; n = 560); (2018; n = 321)] were available for genetic analysis of *pfdhfr* and *pfdhps* loci. A total of 846 samples [(2009; n = 57); (2010; n = 81); (2011; n = 24); (2013; n = 22); (2014; n = 73); (2015; n = 70); (2016; n = 237); (2017; n = 175); (2018; n = 107)] passed as mono-infections (Fws > 0.95 or MOI = 1) and were included in genetic analysis across both genes (Table 1).

**Table 1:** Temporal trends in prevalence of SP resistant haplotypes from 2009 to 2018

Gene	Haplotype^a^	Sampling year								
		2009	2010	2011	2013	2014	2015	2016	2017	**2018**
		% (n/N)	% (n/N)	% (n/N)	% (n/N)	% (n/N)	% (n/N)	% (n/N)	% (n/N)	% (n/N)
*pfdhfr*	(N51**I**/C59**R**/S108**N**/I164**L**)									
	NRNI	7.5 (9/120)	9.2 (17/184)	14.5 (8/55)	6.1 (2/33)	20.7 (67/323)	18.5 (33/178)	7.1 (65/920)	16.1 (90/560)	24.6 (79/321)
	ICNI	3.3 (4/120)	2.2 (4/184)	1.8 (1/55)	6.1 (2/33)	1.2 (4/323)	1.1 (2/178)	5.7 (52/920)	4.8 (27/560)	2.2 (7/321)
	IRNI	80 (96/120)	77.2 (142/184)	76.4 (42/55)	66.7 (23/33)	67.5 (218/323)	73.6 (131/178)	79.8 (734/920)	70.7 (396/560)	67.9 (218/321)
	NCSI	9.2 (11/120)	11.4 (21/184)	7.3 (4/55)	21.1 (7/33)	10.5 (34/323)	6.7 (12/178)	7.5 (69/920)	8.4 (47/560)	5.3 (17/321)
*pfdhps*	(S436**A**[**C**/**F**/**Y**]/A437**G**/K540**E**/A581**G**/A613**S**)									
	(A/F/Y/S)AKAS	14.2 (17/120)	19.0 (35/184)	18.2 (10/55)	6.1 (2/33)	7.1 (23/323)	3.4 (6/178)	3.4 (31/920)	4.1 (23/560)	6.9 (18/261)
	(C/S/A)GKAA	2.5 (3/120)	33.7 (62/184)	9.1 (5/55)	39.4 (13/33)	55.4 (179/323)	64.0 (114/178)	71.2 (655/920)	69.5 (389/560)	78.2 (204/261)
	AAKAA	55.0 (66/119)	22.8 (42/184)	41.8 (23/55)	21.2 (7/33)	21.7 (70/323)	7.3 (13/178)	2.2 (20/920)	9.8 (55/560)	8.4 (22/261)
	(A/S)GKAS	2.5 (3/120)	3.8 (8/184)	3.6 (2/55)	6.1 (2/33)	2.5 (8/323)	9.6 (17/178)	4.6 (42/920)	8.0 (45/540)	4.2 (11/261)
	(S/A)GEAA	0 (0/120)	0 (0/184)	0 (0/55)	3.0 (1/33)	0 (0/323)	1.7 (3/178)	1.1 (10/920)	1.3 (7/560)	0 (0/261)
	SAKAA	25.8 (31/120)	20.7 (38/184)	27.3 (15/55)	24.2 (8/33)	13.3 (43/323)	14.0 (25/178)	17.5 (161/920)	7.3 (41/560)	2.3 (6/261)
*pfdhfr/pfdhps*^*b*^										
	Triple	3.5 (2/57)	0 (0/81)	0 (0/24)	0 (0/22)	5.5 (4/73)	1.4 (1/70)	1.3 (3/237)	0 (0/175)	2.8 (3/107)
	Quadruple	43.9 (25/57)	49.4 (40/81)	87.5 (21/24)	59.1 (13/22)	45.2 (33/73)	25.7 (18/70)	10.1 (24/237)	21.1 (37/175)	20.6 (22/107)
	Quintuple	52.6 (30/57)	50.6 (41/81)	12.5 (3/24)	40.9 (9/22)	49.3 (36/73)	72.9 (51/70)	88.6 (210/237)	78.9 (138/175)	76.6 (82/107)

^a^Haplotypes derived from only pure samples. Quintuple are detected in all three studies and prevalence is much higher when heterozygous samples are included

^b^Triple = NRNI + AAKAA; quadruple = IRNI + SGKAA or IRNI + AAKAA; quintuple = IRNI + AGKAA or IRNI + SGEAA; sextuple = IRNI + SGEGA

### Clinical and demographic characteristics

**Fig. 2:**
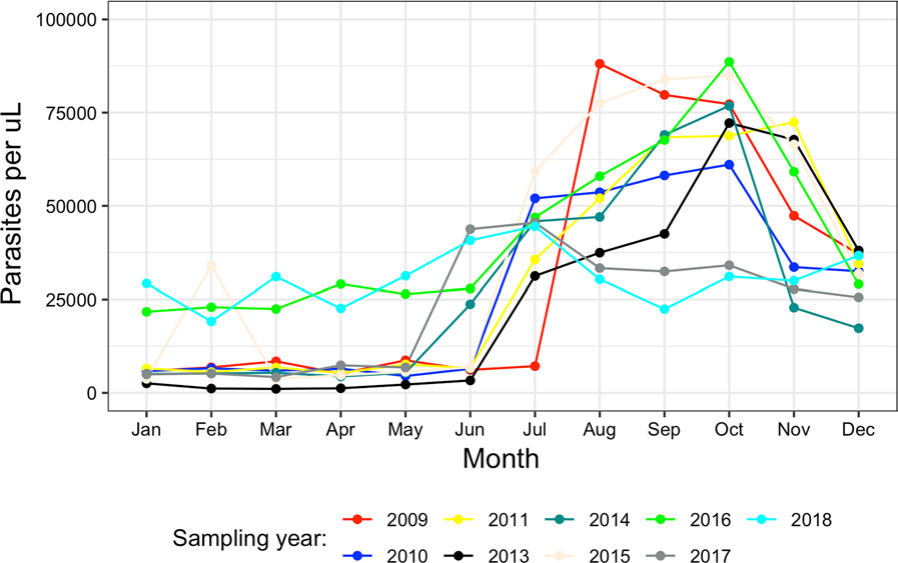
Monthly trends in mean *P*. *falciparum* parasite density from 2009 to 2018

In the years 2009 to 2018, participants were aged between 3.7 to 21.5 years. Overall, there were more female patients (56.3%) than males in the study period 2009 to 2018. The results show a declining trend in parasite density from 2009 to 2018. Trends in monthly mean parasite densities from 2009 to 2018 show a similar unimodal peak during the high malaria season (rainy months of July to November) (Fig. 2 and Additional file 1: Table S1) (Additional file 2: Fig S1).

The median parasite density decreased fourfold from 38,280 (IQR; 62,320) in the 2009–2011 study period to 10,600 (IQR; 52,780) in the 2013–2015 study period but increased to 17,680 (IQR; 49,680) in the 2016–2018 period with a significant decline in the distribution of parasite density during peak transmission from the 2009–2011 study period and the two subsequent surveys (2009–2011 vs 2013–2015 (P < 0.001) or 2016–2018, P < 0.001) (Additional file 3: Fig. S2). Overall, the majority (> 97.8%) of infections were *P. falciparum* mono infections with 2.2% being co-infections with *Plasmodium malariae* as determined from amplicon sequencing data.

### Within-host diversity

Within-host diversity in the earlier studies from 2009 to 2013 ranged from 0.003 to 0.864 (mean 0.254 ± 0.247 SD), estimated by the inbreeding co-efficient *Fws*. Overall, 79.5% (198/249) had *Fws* ≤ 0.5 in the 2009–2013 studies, indicating a high proportion of multi-genomic infections with high potential for outcrossing. Interestingly, overall, in the aggregated data, infections were significantly more complex in the low malaria transmission season (P = 0.022) (Additional file 4: Fig. S3A) but the year on year showed significantly higher diversity only in 2011 (P < 0.001) (Additional file 5: Fig. S4A). In the latter studies from 2014 to 2018 infection complexity ranged from one to three clones, except in 2015 when up to four clones were detected (Additional file 5: Fig. S4B*)*. A higher proportion of monoclonal infections was observed in the 2016–2018 studies in both seasons.

### Temporal trends in *pfdhfr, pfdhps* and the combined *pfdhfr/pfdhps* genotypes

**Fig. 3:**
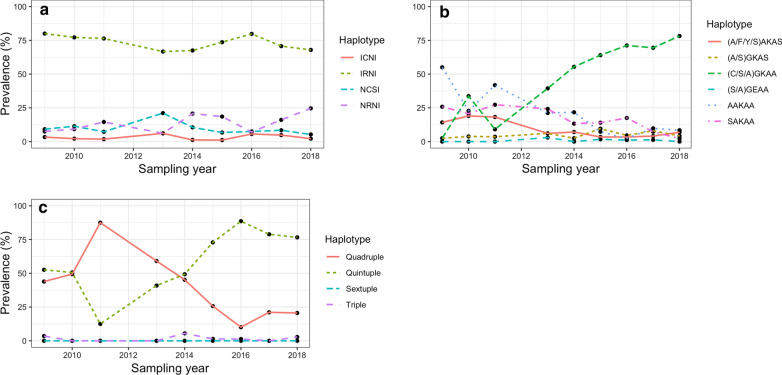
Temporal trends in haplotype prevalence in SP resistance genes from 2009 to 2018. **Panel A**: *pfdhfr* double (**I**C**N**I or N**RN**I*),*triple (**IRN**I) and sensitive (NCSI)*,* **Panel B**: *pfdhps* triple ([**A/F/Y]** AKA**S or [A/**S**]G**KA**S or [**S**/AGE**AA), double (**C**/**S**/A/**G**KAA), single (**A**AKAA) and fully sensitive (SAKAA). **Panel C**: *pfdhr/pfhps* combined haplotypes as defined in Table 1. Mutant amino acid substitutions are shown in bold

The temporal trends in the *pfdhfr*, *pfdhps* haplotypes and *pfdhfr/pfdhps* combined haplotypes were examined on a year-on-year analysis of fluctuations in the prevalence of circulating haplotypes in these genes as shown in Fig. 3. At the *Pfdhfr* locus, year-on-year prevalence of triple (**IRN**I) *Pfdhfr* mutants associated with pyrimethamine resistance is on a general decline though still high (> 60%) (Fig. 3a). Two double mutant haplotypes (N**RN**I and **I**C**N**I) were detected, with haplotype N**RN**I having a higher year-on-year prevalence compared to haplotype **I**C**N**I, except in 2013 when their prevalence estimates were similar (Fig. 3a). The annual prevalence of N**RN**I fluctuated with time from 7.5 in 2009 to 25% in 2018 whilst prevalence of **I**C**N**I fluctuated over time from 3.3% in 2009 to 2.2% in 2018. In general, seasonality analysis showed no significant differences in the prevalence of *pfdhfr* mutants between the wet (high) and dry (low) seasons (Fig. Additional file 6: S5). However, the *pfdhfr* triple mutants increased in prevalence (> 75%) from 2015 to 2016 (when SMC was introduced) during the dry season and fluctuated sharply to about 50% and 59% in 2017 and 2018 respectively (Additional file 6: Fig. S5A). In the dry season comparing the double mutants (NRNI and ICNI), NRNI had about a 20-fold higher prevalence (≤ 20%) than the ICNI mutant (< 1%) between 2011 to 2015 when prevalence of NRNI decreased and that of ICNI increased slightly to narrow their prevalence difference in 2016, after which NRNI increased sharply to > 30% whilst ICNI decreased gradually to < 2% in 2018. On the other hand, during the wet season, whilst *pfdhfr* triple mutants declined similarly as observed in the dry season, albeit a gradual decrease after 2016, the double mutant (NRNI) fluctuated more rapidly at between 3 to 20% from 2009 to 2016 when prevalence of the two mutants equalized to about 2.5% (Additional file 6: Fig. S5B). Between 2016 to 2018, NRNI increased sharply to about 20% whilst ICNI decreased to below 1%. The I164L amino acid substitution associated with high SP treatment failure in some settings was not detected in the current study.

At the *pfdhps* locus, six different haplotypes were observed. Of these haplotypes 5 declined in prevalence over time from between 12.5 and 56% to less than 12.5% with the exception of the *pfdhps* double mutant (**C**/S/**AG**KAA), which fluctuated between 2009 and 2011 and increased consistently from about 9% in 2011 to 71% in 2016, decreased slightly to 69.5% and then increased to about 78% in 2018 (Table 1 and Fig. 3b). The results from seasonality analysis showed a similar trend with the same 5 haplotypes occurring in both seasons and showing a general decline from 2009 to 2018, particularly after 2014 with haplotype ((A/S)GKAS) having higher prevalence in dry season compared to the wet season. However, the double mutant (**C**/S/**AG**KAA), increased in prevalence sharply in both seasons rising from about 33% in 2014 to a peak of about 84% during the dry (low) season in 2016 and then decreasing to 69% in 2018. The double mutant also rose sharply from 46% in 2014 to peak at 85% 2016 and decrease to 82% in 2018 (Additional file 7: Fig. S6B).

Figure 3c shows the yearly fluctuations in the combined *pfdhfr/pfdhps* genotypes. Of the three haplotypes observed (i.e. triple, quadruple and quintuple), the prevalence of triple (N**RN**I + **A**AKAA) haplotype was generally low (0 to 6%) from 2009 to 2018. The quadruple (**IRN**I + **A**AKAA) mutants increased in frequency from 2009 (44%) to 2011 (88%) [pre-IRS] and decreased rapidly from 2011 to about 10% in 2016 before increasing sharply to about 21% in 2017 and 2018 (Table 1 and Fig. 3c). However, prevalence of parasites carrying quintuple mutations (**IRN**I + **AG**KAA or S**GE**AA), which are highly refractory to SP initially decreased from 2009 (53%) to 2011 (13%) and then increased rapidly over time to 77% in 2018 with a light decrease in prevalence between 2016 (89%) and 2017 (79%) (Fig. 3c). No sextuple mutants were detected from 2009 to 2018. The *pfdhfr/pfdhps* combined genotypes did not differ in prevalence between seasons (p > 0.05) but within season prevalence estimates were statistically significant (Wilcoxon, dry season; p < 0.001 & wet season; p = 0.006) (Additional file 8: Fig. S7A). Also, only the prevalence of quintuple haplotype was significantly different between pre-SMC (2009–2015) and post-SMC (2016–2018) (Additional file 8: Fig. S7B). However, the year-on-year dry season prevalence of the quadruple mutants increased sharply from 2009 (9.8%) to 2011 (65%) but rapidly decreased from 2013 (60%) to 2016 (10%) before increasing slightly to 19.8% in 2017 and falling back to 10% in 2018 (Additional file 9: Fig. S8A). The wet season dynamics showed marked fluctuation in the prevalence of quadruple mutants from slightly over 10% in 2009 to 22% in 2011, and about 18% in 2014 to 14% in 2015 before increasing from about 2.5 to 9% in 2017 and 2018 respectively. No quadruple mutants were detected in 2013 and 2016 during the wet season (Fig. S8B). On the one hand, during dry season, quintuple mutant prevalence fluctuated markedly from 10% (2009), 50% (2010) to 12% (2011), then increased gradually through 22% (2013), 28% (2014) to peak at about 48% prevalence in 2015 and 2016 before fluctuating down to 32% in 2017 and 38% in 2018. However, during the wet season quintuple mutants initially found at 5% prevalence were undetected consecutively in 2010 and 2011 but re-emerged in 2013 at 18% prevalence and increased consistently to peak at 46% in 2017 before dropping to a prevalence of 39% in 2018 (Additional file 9: Fig. S8B).

## Discussion

The current study utilized data from Illumina whole genome sequencing and deep amplicon sequencing to determine SNP haplotypes for SP resistance markers following a stringent SNP calling criteria. This enabled reliable haplotype reconstruction in both single and multi-genome infections by accounting for minor clones and avoiding the ambiguity posed when using the standard methodology or individual genotyping assays on mixed infections as observed in this study [31]. A significant decline in the monthly mean prevalence of parasitaemia was observed between the 2009–2011 studies and the subsequent studies in 2013–2015 or 2016–2018 during the high malaria transmission season. The 2009 to 2011 studies were conducted prior to the deployment of strategic interventions, such as IRS and SMC in 2014 and 2016, respectively. Therefore, the marked decline in prevalence of parasitaemia in the post IRS study period 2016–2018 may be due to the direct impact of this intervention aimed at disrupting transmission. The combined effects of IRS at the beginning of the high transmission season and the deployment of SMC (i.e. 4 rounds) during high transmission may explain these observations. However, the observed significantly increased mean parasitaemia post-SMC (2013–2015 vs 2016–2018) corroborates our observed shift in malaria morbidity to higher age groups, which could be attributed to mass treatment campaigns such as SMC that protect young children from clinical malaria. This highlights unintended epidemiological consequences of such targeted mass treatment campaigns and suggest that older children may also benefit from these campaigns. The high within-host diversity observed in the 2009 to 2013 studies with over 70% of infections having Fws < 0.5 is an indication of transmission levels in the era in these settings that pre-date major interventions such as IRS aimed at transmission reduction. The present study highlights the impact of the combined effects of IRS, long-lasting bed nets and SMC, in driving down multi-clonal infections with about 40% of infections harbouring two clones per year from 2014 to 2018, and less than 5% of infections have 3–4 clones between 2014 and 2018. This is consistent with a drop in annual EIR estimates from previous > 250 infective bites/person/year [32] to about 50 bites/person/year with wide variation particularly during the high malaria season in these settings [27].

In the absence of an alternative drug to SP for IPTp and other intermittent preventive treatment programmes in infants such as SMC, monitoring of resistance markers in endemic populations is crucial. In characterizing SP-associated resistance loci in the current study settings, a persistently high prevalence of the triple-mutant (51**I** + 59**R** + 108** N + **I164) *pfdhfr* haplotype from 2009 to 2018 was found. This haplotype, observed at > 60% prevalence year-on-year from 2009 to 2018, has been previously demonstrated to contribute significantly to SP treatment failure [22]. The fairly stable double mutant haplotypes (N**RN**I and I**CN**I**)** appear to differ in fitness with parasites carrying the N**RN**I haplotypes being more prevalent. No marked seasonality was found in distribution of these haplotypes year-on-year but the prevalence of NRNI was higher in dry (low) season suggesting the acquisition of the haplotype alleles by the infection reservoir during high transmission and subsequent seeding of infections in the dry season. Well-designed community studies are required to ascertain the seasonality of these haplotypes. Of note is the complete absence of the *pfdhfr*-164L allele, which has been reported in parts of East Africa [33], but thus far not in West Africa. The counterpart SP-resistance associated gene locus *pfdhps* also had several mutant haplotypes that have been previously characterized to confer high resistance phenotypes. Six different haplotypes were found in this gene, with two (AGEAA and AGKAS) containing markers of high-grade resistance with AGKAS increasing over time and within seasons in this setting. The haplotype AGEAA, which has the K540E mutation previously shown to cause increased SP resistance is observed at < 5% prevalence year-on-year.

The declining year-on-year fluctuations of *pfdhfr* and *pfdhps* haplotypes detected suggest a slow expansion of these haplotypes in this study settings. This may be driven by high recombination rates, particularly during the high malaria season. The higher complexity of infections observed in the current study attest to high transmission intensity in these settings, which is ripe for frequent recombination resulting in breakdown of these long haplotypes.

Also, temporal trends in prevalence of *pfdhfr/pfdhps* combined genotypes show a fluctuating trend of quintuple and quadruple mutants respectively until 2016 when SMC was deployed in our study communities. Since then, the yearly prevalence of both combined haplotypes began to fluctuate in opposing directions with a slight decrease in quadruple mutant prevalence and a slight increase in quintuple prevalence. It is interesting that the distribution of these combined genotypes was not significant between the wet and dry season but rather within each season (Additional file 8: Fig. S7A). This may be attributed to differences in transmissibility within each season. Only quintuple mutants were significantly different between pre-and post-SMC periods, suggesting a possible additional selection pressure imposed by the SMC campaign. This is supported by the observed increased year-on-year prevalence of quintuple mutants in the wet season (Additional file 9: Fig. S8). The overall survival of parasites carrying quintuple mutants all year round may be attributed to increased fitness in the presence of routine anti-malarials and from prophylaxis under SMC. Previous studies have suggested that childhood intervention campaigns such as SMC are likely to promote the spread of resistance in high transmission settings than other adult interventions, such as IPTp-SP [34]. The SMC drugs are given at the beginning of the rainy season (high transmission) in four rounds from July to October. These trends provide support for targeted interventions such as SMC to take place during the high transmission season in order to maximize the long acting effect of AQ partner drug in this intervention to kill these parasites that may have a higher fitness in the presence of SP. However, sentinel data from 2015 to 2017 showed PCR-corrected AS-AQ treatment efficacy of 98.2% in these settings [35], and there is also accumulating evidence of rising prevalence of AQ resistance markers in this population (data to be described elsewhere). Previous studies show that IPTp-SP may remain efficacious in the phase of circulating high grade resistance genotypes but additional mutations, such as 581G, 540E and 613S see the benefits of IPTp-SP begin to decline. Therefore, these mutations may have distinct impacts on different malaria interventions (eg SMC, IPTp among others) and the genotype–phenotype relations may not be perfect. Other factors, such as amplification of parasite GTP cyclohydrolase 1 (*GCH1*) gene that mediate de novo folate biosynthesis [36], host immunity and metabolism may limit these genotype–phenotype interactions.

Overall, barring any sampling bias due to the passive case detection approach in this study, these findings signal a real threat to obtaining the full benefit of key SP-based interventions, such as IPT_p_ and SMC in this setting. However, it is worth noting that the prevalence levels obtained in the current study fall below the WHO limits of > 50% prevalence of 540E to signal failure of SP interventions at a particular setting [22].

Preventing malaria in vulnerable groups such as pregnant women and young children comes with enormous benefits, yet there is currently no effective alternative to SP for use in interventions targeting these groups. Hence, the need to continue to monitor these resistance markers in the general population and target populations to provide evidence-based guidance for the implementation of SP intervention programmes in Ghana. This study, however, is limited to the catchment area of the Navrongo War Memorial Hospital, which is only one out of the 10 NMCP sentinel sites for monitoring anti-malarial drug efficacy across three transmission zones in Ghana [37]. Therefore, there is the need to assess the prevalence of these molecular markers across all 10 sentinel sites with ecological variance across Ghana using targeted sequencing methods to make the data more valuable to the National Malaria Control Programme.

## Conclusion

This study highlights high prevalence of parasites carrying *pfdhfr* triple mutants and rising prevalence of combined *pfdhfr/pfdhps* quintuple mutants in this setting. Thus, indicating the need to continuously monitor parasite response to SP across all three transmission zones in Ghana.

## Supplementary Information

**Additional file 1: Table S1.**

**Additional file 2: Figure S2.** Mean monthly *P. falciparum* parasitaemia from 2009 to 2018.

**Additional file 3: Figure S1.** Distribution of parasite density by intervention period. 1; is 2009–2011 (pre-IRS) study, 2; is 2013–2015 study (IRS) and 3; is 2016–2018 study (SMC). High coverage of long-lasting insecticidal bed nets from 2013.

**Additional file 4: Figure S3** Overall seasonal distribution of *Plasmodium falciparum* complexity of infections. Panel A: Genome-wide *Fws* metric from sequenced data (2009–2013). Panel B: Complexity of infection scored using *COIL* for amplicon data (2014–2018) studies. Study period 1-high season and 2-low season.

**Additional file 5: Figure S4** Annual Seasonal distribution of *Plasmodium falciparum* complexity of infections from 2009–2018. Panel A: Genome-wide *Fws* metric from sequenced data (2009–2013). 1-low season and 2-high season. Panel B: Complexity of infection scored using *COIL* for amplicon data (2014–2018) studies.

**Additional file 6: Figure S5** Seasonal trends of *pfdhfr* haplotypes from 2009 to 2018. Panel A: Low; dry season (low malaria transmission season); Panel B: High; Wet season (High malaria transmission season).

**Additional file 7: Figure S6** Seasonal trends of *pfdhps* haplotypes from 2009 to 2018. Panel **A**: Low; dry season (low malaria transmission season); Panel B: High; Wet season (High malaria transmission season). Hap1-(A/F/Y/S)AKAS; Hap2 -(C/S/A)GKAA; Hap3 – AAKAA; Hap4 -(A/S)GKAS; Hap5-(S/A)GEAA; Hap6 -SAKAA

**Additional file 8: Figure S7** Distribution of *pfdhfr/pfdhps* combined genotypes during high (wet) and low (dry) seasons and pre-and post-SMC periods.

**Additional file 9: Figure S8** Temporal trends of *pfdhfr/pfdhps* combined genotypes during high (wet) and low (dry) seasons and pre-and post-SMC periods.

## Data Availability

Raw data is available on European Nucleotide Archive (ENA). All data generated is presented within the manuscript and its supplementary files.

## Abbreviations

WHO: World Health Organization
SP: Sulfadoxine-pyrimethamine
RDT: Rapid diagnostic test
NMCP: National Malaria Control Programme
SMC: Seasonal Malaria Chemoprevention
IPTp: Intermittent Preventive Treatment in Pregnancy
LLINs: Long-lasting Insecticidal Nets
IRS: Indoor Residual Spraying
EIR: Entomological Inoculation Rate
SNP: Single nucleotide polymorphism
PCR: Polymerase chain reaction
Pfdhfr: *Plasmodium falciparum* dihydrofolate reductase
Pfdhps: *Plasmodium falciparum* dihydropteroate synthetase
