# Supplementary material for: Temporal evolution of sulfadoxine-pyrimethamine resistance genotypes and genetic diversity in response to a decade of increased interventions against Plasmodium falciparum in northern Ghana
Source: Malar J. 2021 Mar 17;20:152. doi: 10.1186/s12936-021-03693-3 (PMC7968364; doi:10.1186/s12936-021-03693-3)
Supplement: Supplementary file 1 — Additional file 1: Table S1. [file 12936_2021_3693_MOESM1_ESM.docx]

Table S1 Demographic and Clinical Characteristics of participants recruited from 2009 to 2018

| Characteristic | 2009 | 2010 | 2011 | 2013 | 2014 | 2015 | 2016 | 2017 | 2018 |
| --- | --- | --- | --- | --- | --- | --- | --- | --- | --- |
|  | **N = 141** | **N =209** | **N =66** | **N =302** | **N = 414** | **N = 186** | **N =1329** | **N = 640** | **N = 1182** |
| Patient age (yr) |  |  |  |  |  |  |  |  |  |
| Mean (SD) | 3.7 (3.6) | 10.1 (12.1) | 6.8 (6.6) | 13.3 (13.5) | 13.1 (13.6) | 8.5 (10.7) | 11.5 (12.2) | 13.6 (13.7) | 21.5 (23.3) |
| Season |  |  |  |  |  |  |  |  |  |
| Low, n(%) | 66 (46.8) | 87 (41.6) | 18 (27.3) | 177 (58.6) | 196 (47.3) | 73 (39.2) | 632 (47.6) | 286 (44.7) | 171 (14.5) |
| High, n (%) | 75 (53.2) | 122 (58.4) | 48 (72.7) | 125 (41.4) | 218 (52.7) | 113 (60.8) | 697 (52.4) | 354 (55.3) | 1011 (85.5) |
| Species |  |  |  |  |  |  |  |  |  |
| Pf ,n (%) | 141 (100) | 209 (100) | 65 (98.5) | 300 (99.3) | 411 (99.3) | 179 (96.2) | 1285 (96.7) | 621(97.0) | 1148 (97.1) |
| Pf/Pm^1^ , n(%) | 0 (0) | 0 (0) | 1 (1.5) | 2 (0.7) | 3 (0.7) | 7 (3.8) | 44 (3.3) | 19 (3.0) | 34 (2.9) |
| Parasitaemia (/μL) |  |  |  |  |  |  |  |  |  |
| Geometric Mean | 78870.7 | 42385.3 | 50395.8 | 35794.7 | 23483.8 | 51471.4 | 29697.5 | 36202 | 29552.7 |
| 95%CI | 67537.5 to 90203.9 | 37230.3 to 47540.2 | 37570.7 to 63220.8 | 30686.2 to 40903.2 | 19351.2 to 27616.5 | 43987.6 to 58955.1 | 26763.2 to 32631.8 | 34059.4 to 38344.7 | 27498.1 to 31607.3 |

^1^ species identified by Microscopy and/or genotyped by amplicon sequencing; High season coincides with the wet months of July to October and Long season the long dry months of November to June.
