# Supplementary figures and images for: Temporal evolution of sulfadoxine-pyrimethamine resistance genotypes and genetic diversity in response to a decade of increased interventions against Plasmodium falciparum in northern Ghana
Source: Malar J. 2021 Mar 17;20:152. doi: 10.1186/s12936-021-03693-3 (PMC7968364; doi:10.1186/s12936-021-03693-3)

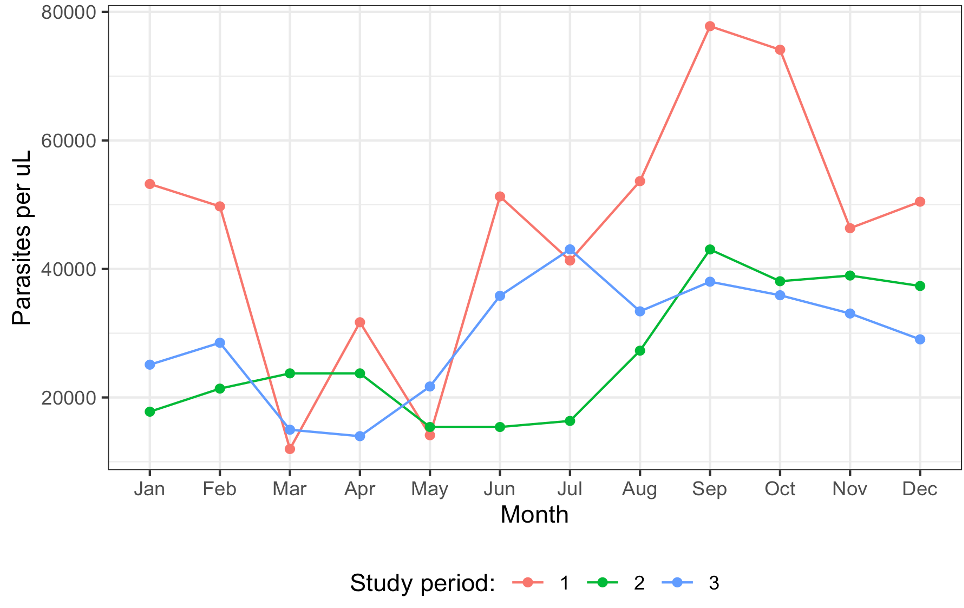

Supplement: Supplementary file 2 — Additional file 2: Figure S2. Mean monthly P. falciparum parasitaemia from 2009 to 2018. [file 12936_2021_3693_MOESM2_ESM.png]

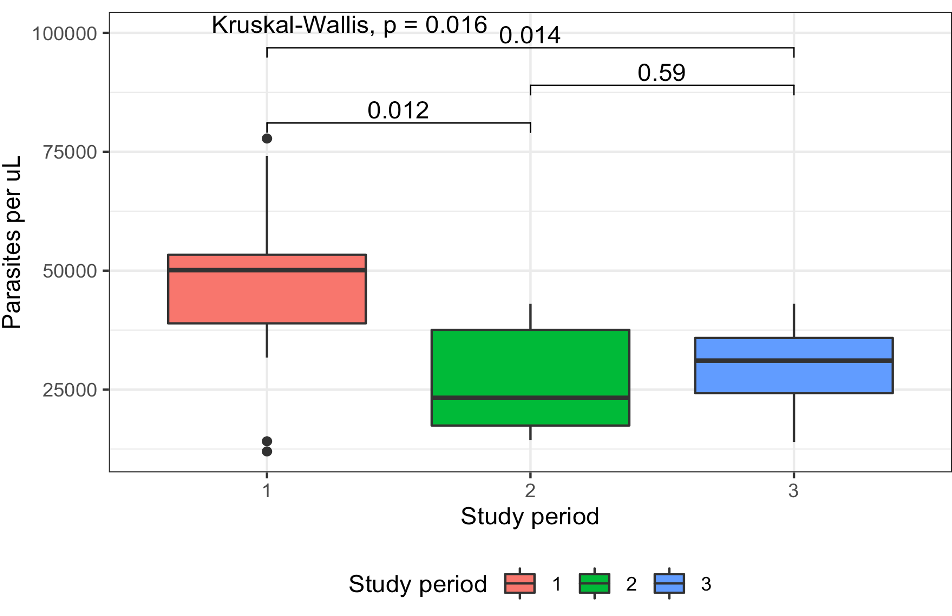

Supplement: Supplementary file 3 — Additional file 3: Figure S1. Distribution of parasite density by intervention period. 1; is 2009–2011 (pre-IRS) study, 2; is 2013–2015 study (IRS) and 3; is 2016–2018 study (SMC). High coverage of long-lasting insecticidal bed nets from 2013. [file 12936_2021_3693_MOESM3_ESM.png]

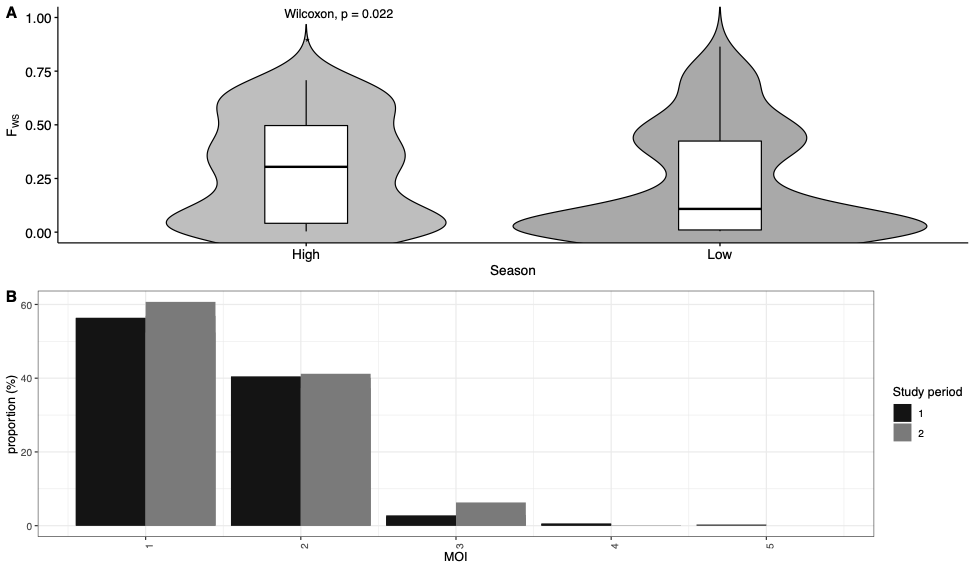

Supplement: Supplementary file 4 — Additional file 4: Figure S3 Overall seasonal distribution of Plasmodium falciparum complexity of infections. Panel A: Genome-wide Fws metric from sequenced data (2009–2013). Panel B: Complexity of infection scored using COIL for amplicon data (2014–2018) studies. Study period 1-high season and 2-low season. [file 12936_2021_3693_MOESM4_ESM.png]

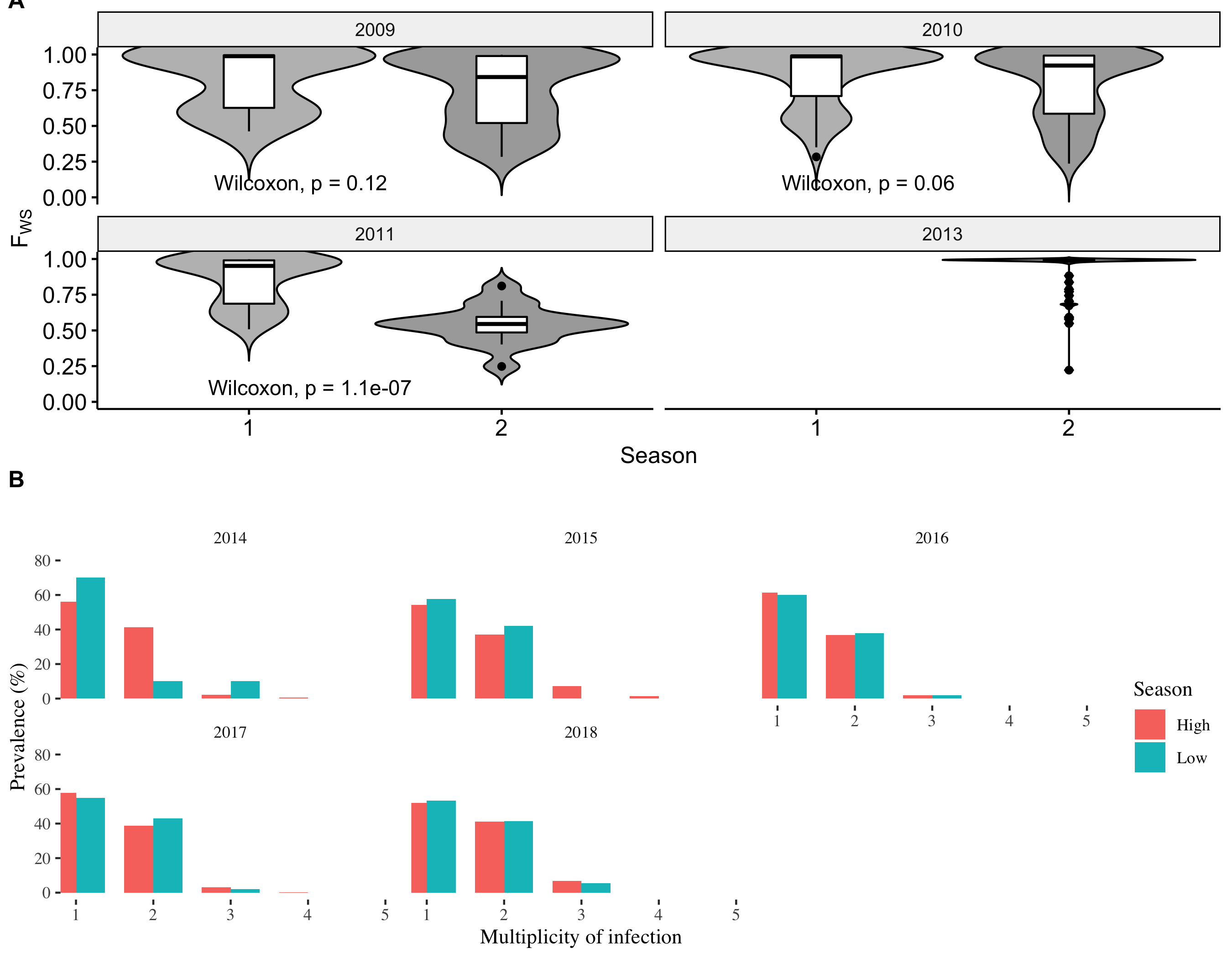

Supplement: Supplementary file 5 — Additional file 5: Figure S4 Annual Seasonal distribution of Plasmodium falciparum complexity of infections from 2009–2018. Panel A: Genome-wide Fws metric from sequenced data (2009–2013). 1-low season and 2-high season. Panel B: Complexity of infection scored using COIL for amplicon data (2014–2018) studies. [file 12936_2021_3693_MOESM5_ESM.png]

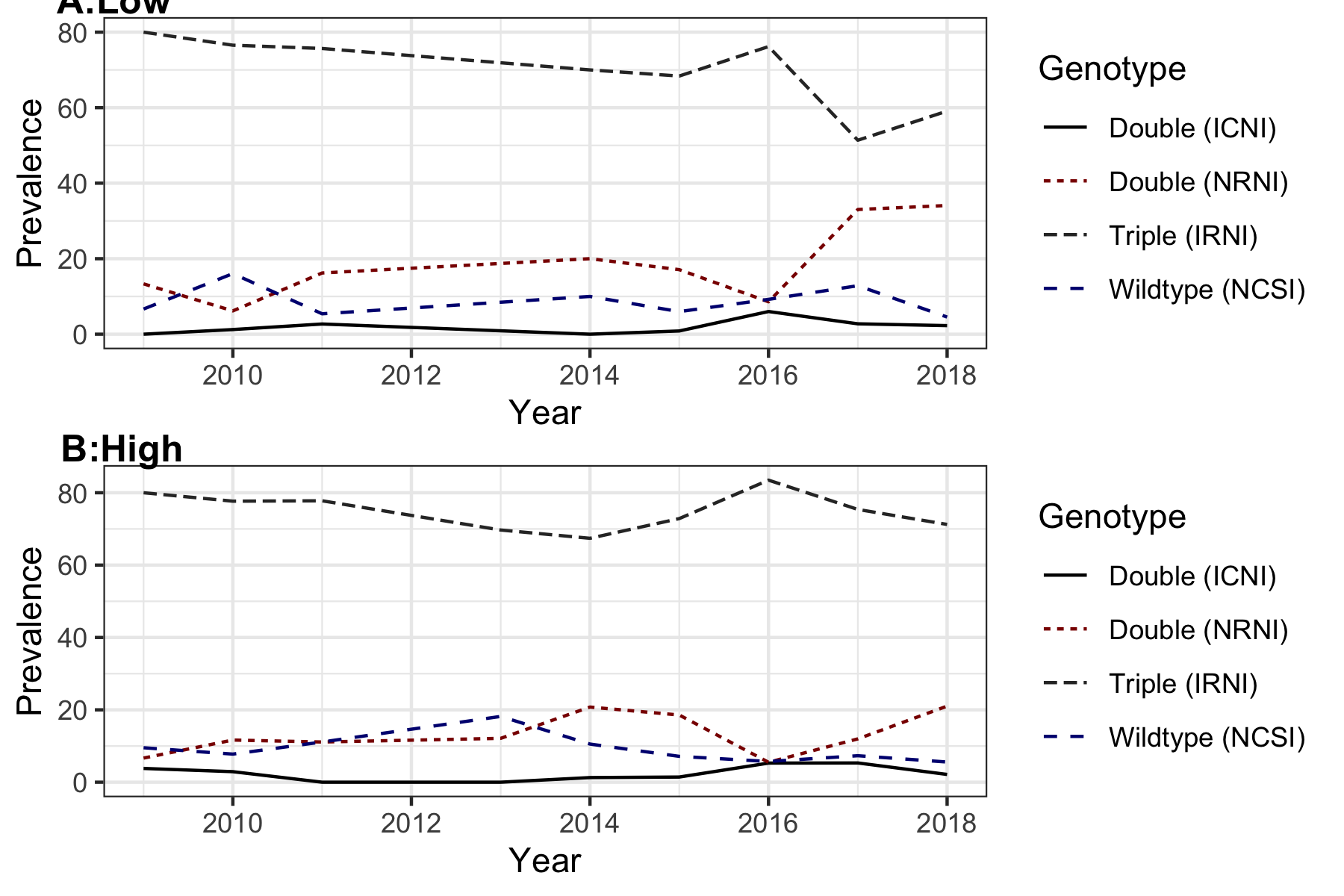

Supplement: Supplementary file 6 — Additional file 6: Figure S5 Seasonal trends of pfdhfr haplotypes from 2009 to 2018. Panel A: Low; dry season (low malaria transmission season); Panel B: High; Wet season (High malaria transmission season). [file 12936_2021_3693_MOESM6_ESM.png]

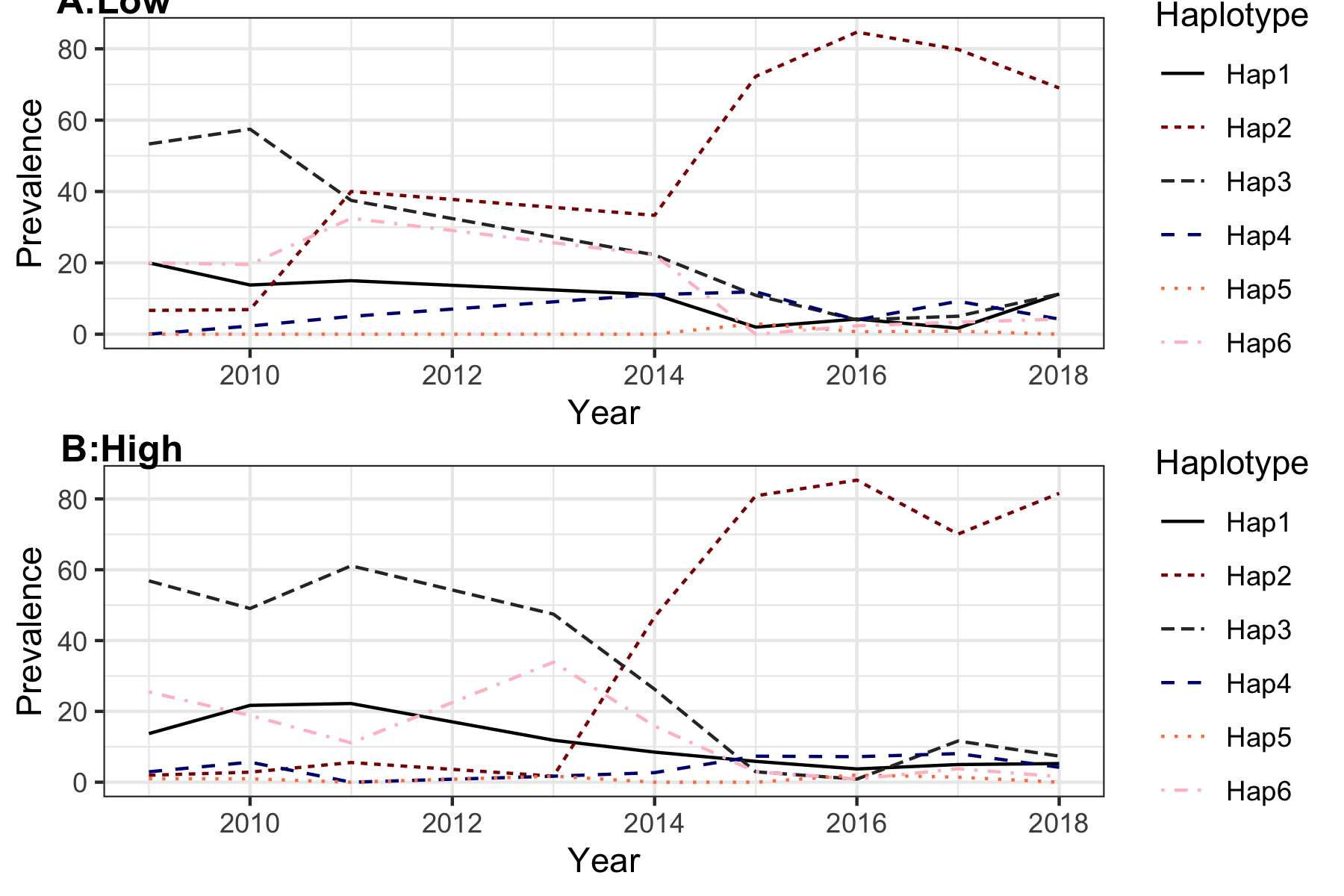

Supplement: Supplementary file 7 — Additional file 7: Figure S6 Seasonal trends of pfdhps haplotypes from 2009 to 2018. Panel A: Low; dry season (low malaria transmission season); Panel B: High; Wet season (High malaria transmission season). Hap1-(A/F/Y/S)AKAS; Hap2 -(C/S/A)GKAA; Hap3 – AAKAA; Hap4 -(A/S)GKAS; Hap5-(S/A)GEAA; Hap6 -SAKAA [file 12936_2021_3693_MOESM7_ESM.png]

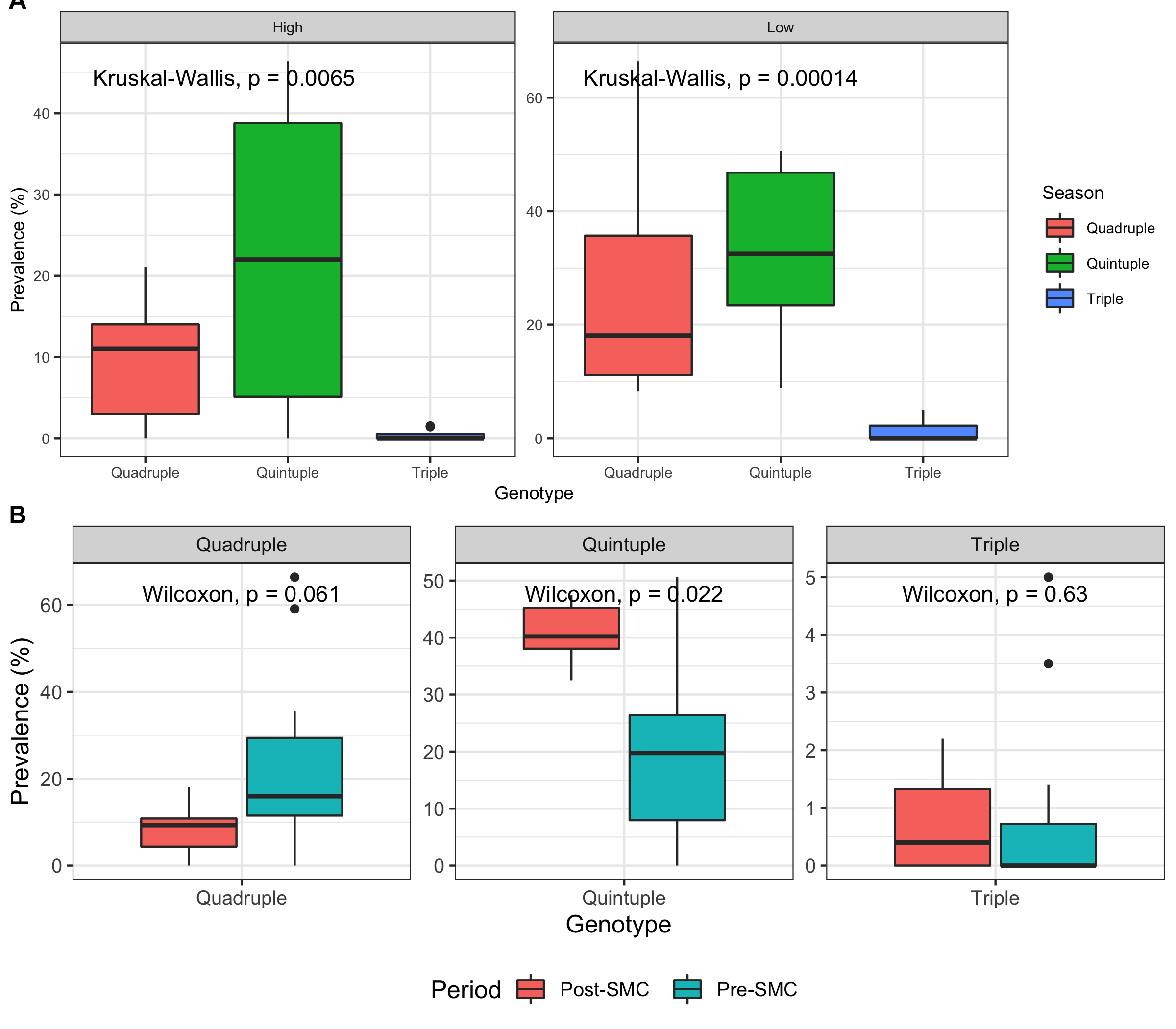

Supplement: Supplementary file 8 — Additional file 8: Figure S7 Distribution of pfdhfr/pfdhps combined genotypes during high (wet) and low (dry) seasons and pre-and post-SMC periods. [file 12936_2021_3693_MOESM8_ESM.png]

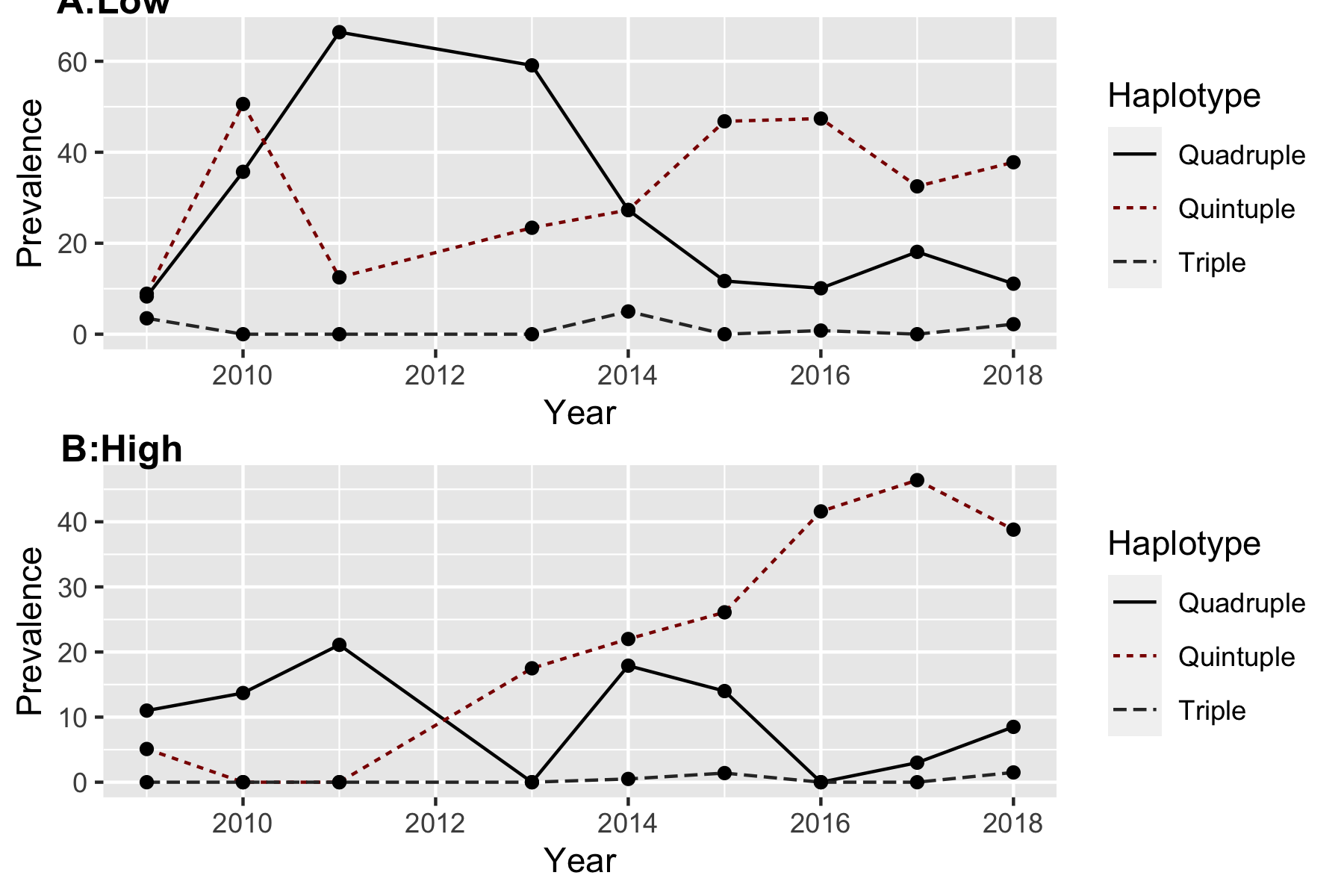

Supplement: Supplementary file 9 — Additional file 9: Figure S8 Temporal trends of pfdhfr/pfdhps combined genotypes during high (wet) and low (dry) seasons and pre-and post-SMC periods. [file 12936_2021_3693_MOESM9_ESM.png]
